# Lean Fish Consumption Is Associated with Beneficial Changes in the Metabolic Syndrome Components: A 13-Year Follow-Up Study from the Norwegian Tromsø Study

**DOI:** 10.3390/nu9030247

**Published:** 2017-03-08

**Authors:** Christine Tørris, Marianne Molin, Milada Cvancarova Småstuen

**Affiliations:** 1 Oslo and Akershus University College of Applied Sciences, Faculty of Health Sciences, NO-0130 Oslo, Norway; Marianne.Molin@hioa.no (M.M.); Milada-Cvancarova.Smastuen@hioa.no (M.C.S.); 2 Institute of Basic Medical Sciences, University of Oslo, Blindern, NO-0317 Oslo, Norway; 3 Bjorknes University College, NO-0456 Oslo, Norway

**Keywords:** metabolic syndrome, insulin resistance, diet, fish consumption, fatty fish, lean fish, processed fish

## Abstract

Background: Fish consumption may have beneficial effects on metabolic syndrome (MetS); however, limited information of such associations exists. This study investigated possible associations between fish consumption and changes in MetS components during a 13-year follow-up period. Methods: The sample included participants (26–69 years) from the Tromsø Study 4 (1994–1995, *n* = 23,907) and Tromsø Study 6 (2007–2008, *n* = 12,981). Data were collected using questionnaires including food frequency questions, non-fasting blood samples, and physical examinations. MetS was defined using the Joint Interim Societies (JIS) definition, in which one point was given for each MetS criteria fulfilled (metabolic score). Longitudinal analyses were performed using Linear mixed models. Results: For both genders, lean fish consumption once a week or more was significantly associated with decreased future metabolic score, decreased triglycerides, and increased high-density lipoprotein (HDL)-cholesterol, whereas decreased waist circumference and blood pressure was identified only for men (age adjusted models). Fatty fish consumption was significantly associated with increased waist circumference for both genders and increased HDL-cholesterol levels in men. *Conclusion*: The results suggest that fatty and lean fish consumption may influence MetS differently and that lean fish consumption in particular seems to be associated with beneficial changes in the MetS components.

## 1. Introduction

Metabolic syndrome (MetS) consists of different risk factors for cardiovascular disease (CVD) and diabetes mellitus type 2 (DM2) [1], and it includes a cluster of metabolic abnormalities such as abdominal obesity, dyslipidemia, hyperglycemia and hypertension. The pathophysiology of MetS is complex and involves insulin resistance, chronic inflammation and ectopic fat accumulation followed by a saturation of the adipose tissue [2]. Abdominal obesity appears to precede the appearance of the other components of MetS [3] and when the adipocytes’ ability to store fat is exceeded, fatty acids are released, resulting in an elevation of circulating free fatty acids (FFA) [4,5]. This increased level of FFA reduces insulin sensitivity in tissue by inhibiting insulin-mediated glucose uptake, which in turn leads to increased circulating blood glucose together with increased pancreatic insulin secretion. The excessive level of circulating insulin, together with the increased level of FFA contributes to hypertension mechanisms, such as enhanced sodium reabsorption [5]. Additionally, sympathetic activity increases in response to the raised insulin, which may cause hypertension in those who are obese [4]. Furthermore, the expansion of adipose tissue leads to an inflammatory response in the fat tissue and to a release of pro-inflammatory cytokines such as tumor necrosis factor alpha (TNF-α) and interleukin 6 (IL-6) [4,5]. The negative impact of MetS on public health is profound, and its rapidly rising prevalence has created an urgent need to identify strategies that may prevent and reverse MetS and its components. 

The beneficial effects of fish consumption on both CVD [6,7,8,9,10,11,12] and DM2 have been described previously [13,14,15]. Since MetS consists of several of the same risk factors as CVD and DM2, one would expect fish consumption to reduce the risk of MetS as well. Lifestyle interventions are the primary strategy in MetS therapy today, and fish consumption could be useful in dietary strategies to improve MetS components. To our knowledge, few studies have investigated associations between fish consumption and MetS [16] and the published results are not conclusive [17,18,19,20,21,22,23,24,25]. 

Fish is an excellent source of protein and other nutrients, such as *n*-3 fatty acids, selenium, iodine, vitamin D and taurine, which may contribute to its health benefits [26]. However, fatty and lean fish contain different amounts of nutrients and there are also some differences in terms of nutrients in wild and farmed fish (Table 1). While fatty fish contain more fat in their tissues and have a larger variety of fatty acids than lean fish, lean fish contain more iodine and less energy than fatty fish [26]. The health benefits of fatty fish are mostly ascribed to their high amount of *n*-3 fatty acids [6]. Lean fish also contain *n*-3 fatty acids, but in contrast to fatty fish, at a smaller extent.

Although the associations between fish consumption and CVD and DM2 have been documented extensively, only limited information on the associations between fish consumption and MetS exists. In particular, there is a lack of studies exploring the possible differences between fatty and lean fish [16]. Therefore, the aim of this study was to investigate possible associations between fish consumption and changes in MetS components using a large population-based sample from Norway during a 13-year follow-up period. Our overall hypothesis was that consumption of fish was associated with beneficial changes in MetS components and thus a healthier metabolic profile.

## 2. Materials and Methods

### 2.1. Materials, Study Design, Settings and Participants

This 13-year follow-up study uses data from the Norwegian Tromsø Study (http://tromsoundersokelsen.no), collected at two time-points. Based on the official population registry, a large representative sample of those living in Tromsø and the surrounding area were invited to participate. The population in Tromsø is mainly made up of Caucasians of Norwegian origin, but also includes a Sami minority population [29]. Initiated in 1974, the Tromsø Study is an epidemiological, population-based study which consists of several cross sectional surveys referred to as Tromsø 1–6 [29,30]. Participants were free to attend any time within each survey data collection period (1 year). The data collection in Tromsø 4 and 6 followed the same design, and consisted of two visits with a basic examination in the first visit and more extensive examinations in the second visit. All examinations, measurements, and laboratory work followed standardized procedures performed by trained health personnel, and the information in the questionnaires was checked by health personnel at the study site [29,30,31].

The participants were 26–69 years old in Tromsø Study 4 in 1994–1995 (*n* = 23,907) and 30–87 years old in Tromsø Study 6 in 2007–2008 (*n* = 12,981). Further, 77% of those who participated in the follow-up in 2007–2008 had also participated in 1994–1995 (*n* = 10,037). The attendance rates were 72% in Tromsø 4, and 66% in Tromsø 6 [29]. Both surveys (Tromsø 4 and 6) included questionnaire data, physical examination and non-fasting blood samples.

The Tromsø Study was approved by the Data Inspectorate of Norway and the Regional Committee of Medical and Health Research Ethics, North Norway [29,31]. Participation in the surveys was voluntary, and each subject gave written informed consent prior to participation [29,31]. This study was approved by the Regional Committee for Medical Research Ethics, South East Norway.

#### 2.1.1. Questionnaires

The questionnaires included both demographic (year of birth, smoking status, education, and leisure time physical activity) and dietary questions (1994–1995) concerning the frequency of lean, fatty and processed fish consumption per week (never, <1, 1, 2–3, 4–5, and approximately every day). In addition, use of cod liver oil/fish oil capsules and vitamin D during the last 14 days (yes, no) was assessed. The nutrients were computed based on the food frequency questionnaire (FFQ) (described elsewhere) [32].

For this follow-up study, the following questions were used:

- How many times per week do you normally eat fatty fish (e.g., salmon/red meat fish) for dinner?
- How many times per week do you normally eat lean fish (e.g., cod) for dinner?
- How many times per week do you normally eat fish balls/fish pudding/fish cakes for dinner?
- Have you taken cod liver oil or fish oil capsules during the last 14 days?
- Have you taken vitamin D supplement during the last 14 days?

Use of cod liver oil/fish oil capsules and vitamin D during the previous 14 days were analyzed as categorical variables (yes, no), while the MetS components were analyzed as continuous variables.

#### 2.1.2. Physical Examination and Blood Samples

To investigate changes in the MetS components, data on MetS components from both Tromsø 4 (1994–1995) and Tromsø 6 (2007–2008) were used. The data collection has been described previously [29,30]. Briefly, the physical examinations included measurements of waist circumference (WC) and blood pressure. WC was measured using a tape measure, without clothing blocking the waist. Blood pressure was measured in a sitting position, using an automated device. Non-fasting blood samples were collected and analyzed for triglycerides (TG), HDL-cholesterol (HDL-C), and blood glucose.

#### 2.1.3. Metabolic Score

The participants were given a score from zero to five for each of the MetS criteria’s fulfilled (abdominal obesity, increased TG, decreased HDL-C, hypertension, and hyperglycemia); thus, a lower metabolic score indicates a better metabolic profile, whereas a higher metabolic score represents a worse profile. In this study, the Joint Interim Societies (JIS) definition of metabolic syndrome is used [1]. The JIS definition recommends different thresholds for WC, to accommodate for differences in various populations and ethnic groups [1]. Here, the International Diabetes Foundation (IDF) cut points for WC were used [33]. Thus, the following criteria for MetS were used: WC ≥ 94 cm in men and ≥80 cm in women, TG ≥ 1.7 mmol/L (150 mg/dL), HDL-C < 1.0 mmol/L (40 mg/dL) in men and <1.3 mmol/L (50 mg/dL) in women, glucose ≥ 5.5 mmol/L (100 mg/dL), systolic blood pressure ≥ 130 mmHg and diastolic blood pressure ≥ 85 mmHg. The blood samples were non-fasting.

#### 2.1.4. Statistical Analyses 

To investigate crude associations between fish consumption and the MetS components, Chi-square tests for categorical variables and analysis of variance (ANOVA) for continuous variables were used, both with fish consumption as a categorical variable (less than once a week/once a week or more) and as continuous variable (never, <1, 1, 2–3 or more). Correlations between pairs of continuous variables were analyzed using Pearson’s correlation coefficients.

Linear mixed models were used to examine changes in the components of MetS and metabolic score, during the 13-year follow-up period, with fish consumption as a categorical independent variable (less than once a week/once a week or more). When examining changes in metabolic score, linear mixed models were fitted with metabolic score (continuous variable) as the dependent variable, with fish consumption (fatty, lean and processed) and time (from 1994–1995 to 2007–2008) as factors (categorical variables), and age (1994–1995) as a covariate (continuous variable) (Model 1). When examining changes in the MetS components, linear mixed models were fitted separately with each of the MetS components modeled as a continuous dependent variable, with fish consumption (fatty, lean and processed), and time (from 1994–1995 to 2007–2008) as factors, and age (1994–1995) as a covariate (Model 1). Further, multiple linear mixed models were fitted with fish consumption (fatty, lean and processed), cod liver oil/fish oil capsules, vitamin D, smoking and time (from 1994–1995 to 2007–2008) as factors, and age (1994–1995), estimated intake of energy and alcohol, education, and leisure time physical activity as covariates (Model 2). In linear mixed models, repeated measurements were available among 4528 participants for WC, 9029 participants for TG, 9020 participants for HDL-C, 9033 participants for BP (SBP and DBP), and 4662 participants for blood glucose.

The *p*-value < 0.05 was considered statistically significant. All tests were two-sided. All analyses were considered exploratory so no correction for multiple testing was performed. All analyses were performed using IBM SPSS Statistics 23 (IBM Corp., Armonk, NY, USA).

## 3. Results

### 3.1. Baseline Characteristics

The baseline characteristics of the participants are presented in Table 2 and Table 3. At baseline (1994–1995), the mean (SD) age of participants (*n* = 23,907) was 44.1 years (11.5), and 48% were men). Mean (SD) body mass index (BMI) was 25.1 (3.8), and 38% reported that they were daily smokers.

Almost 80% of the participants reported lean fish consumption at dinner once or more per week, while 64% reported consuming processed fish and 37% reported consuming fatty fish. Those consuming lean fish once a week or more, were more likely to have a higher level of education, be more physically active, have used cod liver oil/fish oil capsules in the past 14 days, and to be smokers, compared to the fatty fish consumers (Table 2).

Fish consumption increased with increasing age, both for fatty fish consumption and lean fish consumption (Table 3), as did HDL-C, blood pressure, energy intake, dietary fiber, *n*-3 fatty acids and protein. An increase in fatty fish consumption was also observed along with an increase in total fat intake, especially saturated fat. On the other hand, the same association was not observed for lean fish consumption.

### 3.2. Changes during Follow-Up Period

#### 3.2.1. Changes in MetS Components and Metabolic Score during the Follow-Up Period

During the 13-year follow-up period, mean metabolic score increased significantly for both genders. Additionally, several of the MetS components changed significantly: WC, SBP, and blood glucose levels increased significantly for both genders; TG increased significantly for women, and decreased significantly in men; and HDL-C only increased for men. Moreover, a decrease in DBP during the follow-up time was identified; however, this change was only significant in women (Table 4). 

#### 3.2.2. Changes in Metabolic Score by Consumption of Fish during the Follow-Up Period

During the 13-year follow-up period, significantly lower metabolic scores were revealed for those consuming lean fish once a week or more, compared to less than once a week, both in women (B = −0.05, 95% CI −0.09 to −0.01) and men (B = −0.10, 95% CI −0.15 to −0.05) in age adjusted models (Table 5). The associations remained statistically significant after further adjustments in the multiple models. In the age-adjusted models, no significant change in metabolic score was revealed for fatty or processed fish. Interestingly, for those consuming fatty fish once a week or more, compared to those consuming fatty fish less than once a week, an increased metabolic score was observed (B = 0.06, 95% CI 0.02 to 0.10) in the multiple models. The results remained unchanged also after being adjusted for intake of saturated fat.

#### 3.2.3. Changes in MetS Components by Consumption of Fish during the Follow-Up Period

Interestingly, a statistically significant increase in WC was revealed for those consuming fatty fish once a week, compared to those consuming fatty fish less than once a week, during the 13-year follow-up period. This association was identified for both genders, and remained statistically significant in the multiple models. On the other hand, the opposite association was revealed for men who consumed lean fish, with a significant decrease observed in the age-adjusted model. However, this association did not remain statistically significant in the multiple model (Table 6).

Regarding blood pressure, lean fish consumption once a week was associated with a significant decrease in both SBP and DBP, compared to less than once a week, but only for men. However, this finding was no longer statistically significant in the multiple model. For fatty or processed fish consumption, no statistically significant associations were revealed with respect to blood pressure (Table 6).

A significant decrease in TG was observed for those consuming lean fish once a week or more, compared to those consuming lean fish less than once a week, in both genders in age adjusted models; women (B = −0.040, 95% CI −0.008 to −0.002), men (B = −0.111, 95% CI −0.17 to −0.06). However, in the multiple models these associations were significant only for men. Surprisingly, no associations were identified between intake of fatty or processed fish and TG (Table 7).

A statistically significant increase in HDL-C was observed for those consuming lean fish once a week or more, compared to those consuming lean fish less than once a week, both for women (B = 0.03, 95% CI 0.01 to 0.05) and men (B = 0.04, 95% CI 0.02 to 0.05) in the age adjusted models (Table 7). These associations remained significant in the multiple models. The level of HDL-C increased significantly for men consuming fatty fish once a week or more, compared to those consuming fatty fish less than once a week (B = 0.02, 95% CI 0.001 to 0.03), however, this change was not statistically significant in the multiple model. No association between fatty fish consumption and HDL-C was revealed among women.

A significant decrease in HDL-C was observed for those consuming processed fish once a week or more, compared to those consuming processed fish less than once a week, both for women (B = −0.022, 95% CI −0.04 to −0.005) and men (B = −0.024, 95% CI −0.04 to −0.01) in the age adjusted models (Table 7). The association remained significant in the multiple model.

No associations between fish consumption and changes in blood glucose were identified during the 13-year follow-up period (Table 7). The results remained unchanged for all the MetS components also after being adjusted for intake of saturated fat.

## 4. Discussion

In this 13-year follow-up study, lean fish consumption was associated with beneficial changes in four out of five components comprising MetS improvements were identified in abdominal obesity (WC), lipid profile (TG and HDL-C), and blood pressure. However, some of the identified associations were only statistically significant in men (WC and blood pressure).

Several mechanisms may partly explain the beneficial effects of fish consumption on the components of MetS, which presumably work through reduced ectopic fat accumulation, improved lipid metabolism, and hypotensive effects.

### 4.1. Abdominal Obesity

During the 13 years of follow-up, lean fish consumption was associated with a decrease in WC only for men. In contrast, fatty fish consumption was associated with an increase in WC for both genders. Previously, fish consumption has been associated with a decrease in WC in intervention studies, both for those consuming lean fish [25,34,35] and those consuming fatty fish [34], when compared to their controls (no fish or seafood). However, an energy restricted diet (−30%) may have influenced the results in two of the intervention studies [25,34]. Still, a greater decrease in WC was observed in participants consuming cod (150 g) 5× per week (mean difference −3.419, 95% CI −4.830 to −2.007), than those consuming cod (150 g) 3× per week (mean difference −0.994, 95% CI −2.520 to 0.532), when compared to no fish consumption [25]. Furthermore, a decreased WC was reported for participants receiving 100 g per day of lean fish (Namibia hake) with seven servings per week, when compared with no fish consumption [35]. This might suggest a dose-response relationship between lean fish consumption and WC. However, results are conflicting, and not all studies have observed significant associations between fish consumption and a decrease in WC [17,22]. However, in these studies, fatty and lean fish were not assessed separately.

Lean fish, such as cod, is considered a superior source of proteins, and may contribute to the treatment of obesity by acting on metabolic targets such as satiety and energy balance [36,37,38]. However, little is known about possible differences between the proteins in fatty fish versus lean fish. Furthermore, fish protein contains different amino acids, such as taurine, which may have beneficial effects on obesity [39,40]. The highest concentrations of taurine have been found in lean fish [28], Furthermore, a diet rich in fish has been associated with lower plasma leptin [41], a hormone known for the regulation of appetite and fat storage in mammals [42]. Fish has one of the highest natural concentration of iodine out of all foods [43]. Iodine is essential to the production of thyroid hormones that regulate metabolism and the way our body uses energy. The highest values of iodine are found in lean fish such as cod (199–130 mcg per 100 g), whereas fatty fish such as salmon and trout contains less (5–19 mcg per 100 g) [27]. The recommended daily intake (RDI) of iodine for adults is 150 mcg per day [44], which has been considered the appropriate amount to allow for normal T4 production without burdening the thyroid [45]. Furthermore, several of the nutrients in fish, such as proteins, taurine and *n*-3 fatty acids, have been associated with an anti-inflammatory effect, possibly through their effect on cytokines such as TNF-α [39,46,47,48].

### 4.2. Improved Lipid Profile

In this follow-up study, lean fish consumption once a week or more was associated with an improved lipid profile with a decreased TG and an increased HDL-C for both genders, when compared to lean fish consumption less than once a week. In addition, fatty fish consumption once a week or more was associated with an increased HDL-C, however, only in men. In contrast, consumption of processed fish was associated with significantly decreased HDL-C in both genders. A significant increased HDL-C was revealed in men but no change in women was found from baseline to follow-up. In this study, women consumed more processed fish, compared to men, and this might play a role in this gender difference found regarding HDL-C. However, several other confounding factors such as menopause could be of importance here [49,50].

In previous studies, higher fish consumption has been associated with an increased HDL-C in both men [17] and women [22]. However, when fatty fish consumption and lean fish consumption were examined separately in intervention studies, a significant increase in HDL-C was reported for fatty fish [51,52], but not for lean fish [52]. Fish consumption has also been associated with a decreased TG both in women [22,53], and in men [17]. Still, the results are conflicting, and not all studies have found such associations [36,54]. Nevertheless, in randomized controlled trials, both lean fish [52,55] and fatty fish have been associated with a decreased TG [51,52], when compared to non-seafood groups. On the other hand, higher TG levels have also been reported in those with higher fish consumption, compared to no fish consumption [23]. Interestingly, we previously reported that fish consumption once a week or more was significantly associated with higher TG in women and a lower TG in men, when investigating fatty and lean fish together [21]. 

Cod protein has been associated with beneficial effects on lipid metabolism in rats [56], and beneficial effects of taurine on lipid profiles have also been suggested [39,40,57]. Dietary proteins have been found to slow both the absorption and synthesis of lipids, and to promote lipid excretion [58]. However, these mechanisms may differ depending on the protein sources. Recently, a higher lipid catabolism after consumption of lean seafood was suggested [59]. 

In this follow-up study, a higher proportion of the lean fish consumers reported using cod liver oil/fish oil capsules, compared to the fatty fish consumers. However, in the multiple models this was adjusted for (cod liver oil/fish oil capsules use). Consumption of fish oil has been associated with decreased TG [60,61,62,63] as well as increased HDL-C [61,64,65]. *N*-3 fatty acids are considered a valuable clinical tool in treatment of hypertriglyceridemia [66], and it has been argued that *n*-3 fatty acids may be more efficacious when consumed in fish rather than when an equivalent amount is provided by fish oil capsules [67]. 

Interestingly, our data revealed a decrease in HDL-C in those consuming processed fish once a week or more. In Norway, processed fish such as fish balls, fish fingers, fish pudding and fish cakes are commonly made out of lean fish filet mixed with other ingredients such as flour and milk, and these fish products represent approximately 40% of the total fish consumption [68]. The lack of health benefits from processed fish may partly be explained by a reduction of some of the nutrients present during the processing (deep-fried, fried, boiled or minced). For instance, the loss of taurine in processed products may be as high as 100%, compared to the taurine content of fresh caught specimens [69]. Furthermore, ultra-processed fish, such as fish fingers, are deep-fried and may therefore contain a higher amount of total fat. Additionally, such products previously contained trans-fatty acids, a fatty acid known to be associated with lowered HDL-values [70], when the data from Tromsø 4 was collected (1994–1995). We therefore cannot rule out the possibility that the lowered HDL found after intake of processed fish in the present study may be influenced by the trans-fatty acids contained in such products.

### 4.3. Hypotensive Effects

In the present study, lean fish consumption was associated with decreased blood pressure—both SBP and DBP decreased significantly during the follow-up period. However, in the age-adjusted models, this effect was only confirmed in men. In contrast, blood pressure increased during follow-up for those consuming fatty or processed fish, although this finding was not statistically significant. Previously, fish consumption has been associated with decreased blood pressure in various intervention studies [35,51,54,71]. However, the results are conflicting—some studies found associations for lean fish, and others for fatty fish. In line with our study, a decrease in blood pressure (SBP and DBP) was reported in participants consuming lean fish (pike, pike-perch, perch, saithe, cod), while blood pressure increased among those consuming fatty fish (salmon, rainbow trout, baltic herring, whitefish, vendace, tuna), in an 8-week intervention study from Finland [54]. A Spanish randomized crossover clinical trial (*n* = 273) also found lean fish consumption to have a blood pressure lowering effect, when participants were given seven servings of lean fish per week (Namibian hake). However, this finding was significant only for DBP [35]. In contrast, a decrease in blood pressure (SBP and DBP) has also been reported after fatty fish consumption (125 g per day of salmon for four weeks), compared to controls (no fish) [51]. 

It has previously been suggested that fish protein lowers blood pressure in rats, when compared to rats given casein [72], and amino acids, such as taurine, may improve blood pressure by influencing the Angiotensin II action [40] and have a hypotensive effect [39]. Previously, beneficial effects of fish proteins on insulin actions have been observed. In a randomized controlled trial, improved insulin sensitivity was found in participants in the cod protein group, when they were compared to those receiving other animal proteins (lean beef, pork, veal, eggs, and milk products) [73]. In addition, fish proteins have been observed to have beneficial effects on both insulin sensitivity and insulin resistance in rats [48,74].

### 4.4. Blood Glucose

In the present study, no association was observed between fish consumption and change in blood glucose. This is in line with other studies [17,54]. However, in this follow-up study, blood serum samples were non-fasting which may have influenced the glucose results.

### 4.5. Gender Differences

In this follow-up study, the association between lean fish consumption and reduced WC was found only in men. Such gender differences could be explained by differences in body-fat distribution, lipid levels, as well as sex hormones. Compared to men, women have a higher percentage of body fat with more adipose tissue on the hips and thighs, and they may also accumulate more adipose tissue without metabolic consequences, particularly in the gluteal-femoral tissue [75]. Furthermore, the amounts of sex hormones differ between the genders, in addition to changes in hormone levels that occur over an individual’s lifetime. Both testosterone and estrogen levels have been associated with components of MetS [76], and ovarian hormones, such as estrogen, have been suggested to have a protective role regarding MetS, at least before menopause [77]. This effect may be due to the ability of estrogen to decrease inflammation and reduce the glucocorticoid response [78]. In addition, estrogen may have a protective effect on adipose tissue—the hormone’s anti-inflammatory effects may protect women from diseases associated with the inflammation of adipose tissue [79]. Men and women also regulate energy balance differently, and estrogen may directly influence energy balance through its regulation of food intake and body adiposity [77]. 

Gender differences in lipid profile have previously been observed, e.g., women at all ages seems to have higher HDL-C than men [76]; as reflected in the MetS definition [1]. In addition, aging and menopause may lead to differences in lipid profiles [76] and an increase in TG level has been reported with an increasing age for both genders [76].

### 4.6. Contaminants and Other Undesirable Substances in Fish

Fish consumption has many positive health effects, however, fish may also contribute to dietary exposure to contaminants and other undesirable substances such as methylmercury, dioxins and dioxin-like polychlorinated biphenyls (PCBs) [26].

Marine fat is a major dietary exposure source of dioxins and PCBs and fatty fish is considered to be the main source, contributing to 76% of the exposure from fish [26]. Dioxins and PCBs comprise a subgroup among the persistent organic pollutants (POPs), found in the highest concentrations high up in the food chain [26]. Exposure to POPs may lead to increased levels of serum lipids and dyslipidemia [80], and therefore may be involved in the pathogenesis of MetS. Furthermore, PCBs have been associated with a higher risk of hypertension [81], and dioxin-related compounds in particular have been suggested to increase the risk of hypertension [82]. Mercury has also been associated with an increased risk of hypertension [83], however, the possible harmful effects of mercury may be attenuated by high levels of selenium [84].

Moreover, farmed fish tend to have higher lipid levels than wild fish, which can lead to higher bioaccumulation of lipophilic persistent pollutants, and higher levels of some organic contaminants have been found in aquaculture products, compared to samples from extractive fishing [85]. Nevertheless, the benefits of fish consumption have been found to outweigh any negligible risk presented by contaminants and other known undesirable substances in fish [26].

### 4.7. Strengths and Weaknesses

To the best of our knowledge, this is the largest conducted study with the longest follow-up period exploring fish consumption and MetS. The main strength of this study is the high number of participants, recruited from a homogenous population from large population-based surveys conducted at two time points. The standardized procedures used by trained health personnel in examinations, measurements, and laboratory work increases the validity and reliability in the study [29]. Further, the attendance rates were high (72% in Tromsø 4 and 66% in Tromsø 6) [29], and 77% of those who participated in 2007–2008 also participated in 1994–1995. 

However, despite the high number of attendees and high attendance rate, the results from this follow-up study should be regarded as hypothesis-generating, and should be tested further in other studies, especially to clarify the role of lean vs. fatty fish in the risk of MetS. Additionally, the original blood serum samples were non-fasting and this may have influenced the results, which might have led to a somewhat higher MetS score for some individuals. However, if there is a bias in the estimates due to non-fasting measurements, then this bias is the same for all the individuals included in our sample. Our goal was to investigate associations between fish consumption and MetS components, especially regarding any possible differences between lean and fatty fish. Further, we have no reason to believe that the non-fasting blood samples were measured differently in the different fish consumption groups. Therefore, if the proportions of those with MetS score were overestimated, this overestimation is present and equally distributed in both groups thus the comparisons between groups are still valid. In addition, we performed a sensitivity analysis. We assumed that all blood samples were non-fasting and therefore increased the glucose criteria to ten or larger, and the results remained the same thus confirming our statement above. 

The main weakness of this study is that there is an overlap among those consuming fatty, lean, and processed fish, due to the high overall consumption of fish in this sample and the limitation in the food frequency questionnaire. Therefore, additional effects from consuming every category of fish cannot be ruled out. In addition, only fish consumption at dinner was assessed, and possible additional effects from fish consumption in other meals, such as lunch, cannot be ruled out. Additionally, the present study uses data on fish consumption frequency and not on portion size. The high level of fish consumption observed in participants may have limited the possibility to investigate effects of intake of smaller amounts of fish, especially regarding total and lean fish. It has been reported that survey participant tend to over-report consumption of healthy foods and under-report consumption of what is perceived as unhealthy food, however, we have no reason to believe that lean fish is more or less often reported than fatty fish or processed fish. High reproducibility has also been observed when using FFQs to assess key nutrients of a healthy Mediterranean diet [86].

## 5. Conclusions

In this large population-based study from Norway, lean fish consumption was associated with beneficial changes in four of the five MetS components during the 13-year follow-up period. Several gender related differences emerged: reduced abdominal obesity (WC), improved lipid profile (TG and HDL-C), and reduced blood pressure were found in men, whereas only an improved lipid profile (TG and HDL-C) was found in women. Interestingly, lean fish consumption was associated with beneficial changes in future metabolic score for both genders. 

In conclusion, these results suggest that consumption of fatty and lean fish may influence metabolic score differently, and that lean fish consumption seems to have a greater beneficial effect on the various components of MetS when compared to fatty fish. Further investigations are warranted to shed more light on the possible role and mechanisms of fatty vs. lean fish consumption in the development of MetS components.

## Figures and Tables

**Table 1:** Nutritional profile of commonly consumed, whole, raw fish, per 100 g [27,28].

Nutrients	Cod		Salmon	
	Wild	Farmed	Wild	Farmed
Energy (kJ)	343	358	760	932
Fat (g)	1.1	0.5	12	16
SFA (g) ^1^	0.1	0.1	1.8	3
MUFA (g) ^1^	0.1	0	4.4	5.9
PUFA (g) ^1^	0.3	0.2	1.9	5
Omega-3 (g)	0.5	0.2	1	1.5
Protein (g)	17.9	20	19.7	20
Taurine (mg)	108	- ^2^	60	60
Fat soluble vitamins				
Retinol (µg)	12	2	0	26
Vitamin D (µg)	2	0.7	8	10
Water soluble vitamins				
Niacin (mg)	1.8	3.9	7	7.3
Vitamin B6 (mg)	0.12	0.26	0.6	0.51
Folate (µg)	11	11	13	7
Vitamin B12 (µg)	1.5	1	6.9	3.5
Minerals and trace elements				
Selenium (µg)	22	30	50	30
Iodine (µg)	119	300	- ^2^	10

^1^ SFA: saturated fatty acids, MUFA: monounsaturated fatty acids and PUFA: polyunsaturated fatty acids; ^2^ No information.

**Table 2:** Baseline characteristics for categorical variables by frequency of weekly fish consumption, %.

	*n*/Total ^1^	Never	Less than Once	Once	2–3 or More	*p* ^1^
Fatty fish consumption						
Females	10,269/19,822	11.2	53.5	29.2	6.0	<0.0001
Males	9553/19,822	9.6	51.6	30.4	8.3	<0.0001
Higher education ^2^	8610/19,773	10.5	57.2	27.4	4.9	<0.0001
Physical activity ^3^	6670/19,717	10.1	53.5	29.7	6.7	0.2
Smoking	7345/19,802	12.1	50.9	29.3	7.7	<0.0001
Cod liver oil ^4^	6283/18,746	8.5	49.5	33.3	8.8	<0.0001
Lean fish consumption						
Females	10,791/20,806	2.5	19.6	48.1	29.9	<0.0001
Males	10,015/20,806	2.7	19.9	45.2	32.2	<0.0001
Higher education ^2^	8910/20,747	3.0	22.8	49.3	24.9	<0.0001
Physical activity ^3^	6958/20,687	2.6	19.8	48.7	28.9	<0.0001
Smoking	7675/20,789	3.1	21.1	45.2	30.5	<0.0001
Cod liver oil ^4^	6590/19,547	1.9	16.3	47.9	33.9	<0.0001
Processed fish consumption ^5^						
Females	10,578/20,210	4.3	26.8	55.1	13.8	<0.0001
Males	9632/20,210	7.3	34.3	49.1	9.4	<0.0001
Higher education ^2^	8762/20,153	6.6	28.9	52.6	11.8	<0.0001
Physical activity ^3^	6799/20,099	6.3	29.7	53.1	11.0	<0.01
Smoking	7460/20,194	6.3	31.9	50.4	11.4	<0.0001
Cod liver oil ^4^	6424/19,106	5.1	29.3	53.5	12.1	0.001

Numbers of participants vary because of missing information variables. ^1^ *p*-value derived using Chi-square tests with fatty, lean and processed fish consumption as categorical variables (never, <1, 1, 2–3 or more); ^2^ Higher education: High school diploma or more; ^3^ Average of three or more hours of vigorous leisure time hard physical activity per week (sweating/out of breath once a week or more) during the last year; ^4^ Cod liver oil/fish oil capsules used past 14 days; ^5^ Fish balls/fish pudding/fish cakes. The Tromsø Study: Tromsø 4.

**Table 3:** Baseline characteristics (1994/1995) for continuous variables by frequency of weekly fish consumption mean (SD).

	*n* ^1^	Never	Less than Once	Once	2–3 or More	*p* ^2^
Fatty fish						
Age (years)	19,822	38.7 (10.7)	42.3 (11.0)	46.5 (11.3)	50.1 (11.3)	<0.0001
BMI	19,798	24.6 (4.0)	24.8 (3.7)	25.3 (3.8)	25.7 (3.8)	<0.0001
WC (cm)	4361	89.1 (12.8)	88.9 (11.2)	89.8 (11.1)	91.8 (11.7)	<0.0001
TG (mmol/L)	19,785	1.53 (1.06)	1.49 (1.00)	1.53 (1.07)	1.57 (1.02)	0.007
HDL-C (mmol/L)	19,767	1.46 (0.39)	1.49 (0.39)	1.52 (0.42)	1.52 (0.42)	<0.0001
SBP (mmHg)	19,806	132.0 (16.4)	133.8 (17.0)	136.5 (18.5)	138.9 (19.4)	<0.0001
DBP (mmHg)	19,806	77.4 (12.0)	78.6 (12.3)	80.4 (13.0)	82.1 (13.6)	<0.0001
Glucose (mmol/L) ^4^	5137	4.9 (1.4)	4.8 (1.1)	4.9 (1.4)	4.9 (1.2)	0.01
Energy (MJ/day)	16,660	7.35 (2.17)	7.87 (2.16)	8.21 (2.22)	8.67 (2.35)	<0.0001
Fibre (g/day)	16,660	18.9 (6.9)	21.3 (7.1)	22.7 (7.0)	23.0 (7.3)	<0.0001
Protein (g/day)	16,660	68.1 (20.1)	76.0 (20.0)	82.1 (20.8)	95.4 (23.8)	<0.0001
Total fat (g/day)	16,660	63.2 (24.7)	66.0 (23.1)	67.8 (23.2)	74.2 (25.9)	<0.0001
*n*-3 FA(g/day)	16,660	0.32 (0.46)	0.65 (0.49)	1.00 (0.62)	1.83 (0.88)	<0.0001
Saturated fat (g/day)	16,660	27.4 (11.2)	27.8 (10.2)	27.9 (10.2)	29.0 (11.0)	0.001
Alcohol (g/day) ^3^	16,660	3.0 (4.2)	3.2 (4.2)	3.5 (4.6)	3.6 (5.2)	<0.0001
Lean fish						
Age (year)	20,806	36.2 (10.0)	39.3 (10.2)	43.5 (10.9)	49.8 (12.2)	<0.0001
BMI	20,778	24.5 (3.9)	24.8 (3.7)	24.9 (3.7)	25.5 (3.9)	<0.0001
WC (cm)	4787	86.9 (13.9)	89.2 (11.9)	88.9 (11.3)	90.5 (11.2)	<0.0001
TG (mmol/L)	20,763	1.58 (1.04)	1.52 (1.05)	1.50 (1.01)	1.54 (1.05)	0.01
HDL-C (mmol/L)	20,743	1.43 (0.41)	1.46 (0.38)	1.50 (0.41)	1.52 (0.41)	<0.0001
SBP (mmHg)	20,790	131.5 (14.9)	132.7 (16.5)	134.0 (17.2)	138.2 (19.9)	<0.0001
DBP (mmHg)	20,790	76.8 (11.0)	77.6 (12.0)	78.8 (12.5)	81.6 (13.3)	<0.0001
Glucose (mmol/L) ^4^	5590	5.1 (2.6)	4.7 (0.9)	4.8 (1.3)	4.9 (4.9)	0.007
Energy (MJ/day)	16,903	7.4 (2.3)	7.6 (2.2)	7.9 (2.2)	8.4 (2.3)	<0.0001
Fibre (g/day)	16,903	17.2 (7.1)	18.9 (6.7)	21.6 (6.9)	23.7 (7.1)	<0.0001
Protein (g/day)	16,903	67.0 (22.2)	71.3 (20.1)	76.5 (19.8)	86.4 (21.6)	<0.0001
Total fat (g/day)	16,903	66.7 (26.2)	66.8 (24.3)	66.1 (22.9)	67.2 (24.0)	0.05
*n*-3 FA(g/day)	16,903	0.34 (0.51)	0.62 (0.53)	0.77 (0.60)	0.96 (0.73)	<0.0001
Saturated fat (g/day)	16,903	29.1 (12.1)	28.2 (10.7)	27.6 (10.0)	27.9 (10.4)	0.001
Alcohol (g/day) ^3^	16,903	3.8 (6.1)	3.5 (4.6)	3.4 (3.4)	2.9 (2.9)	<0.0001
Processed fish						
Age (year)	20,210	42.7 (11.9)	45.0 (11.7)	43.6 (11.3)	42.1 (11.6)	<0.0001
BMI	20,183	25.0 (3.7)	25.2 (3.8)	24.9 (3.7)	24.8 (3.8)	<0.0001
WC (cm)	4485	91.1 (11.6)	90.0 (11.4)	89.1 (11.2)	89.0 (11.8)	0.007
TG (mmol/L)	20,172	1.60 (1.09)	1.55 (1.01)	1.49 (1.03)	1.48 (1.03)	<0.0001
HDL-C (mmol/L)	20,152	1.46 (0.43)	1.49 (0.41)	1.50 (0.40)	1.50 (0.39)	0.003
SBP (mmHg)	20,195	135.1 (17.0)	135.7 (17.9)	134.2 (17.6)	133.7 (17.7)	<0.0001
DBP (mmHg)	20,195	79.6 (12.2)	79.9 (12.7)	78.9 (12.5)	78.3 (12.7)	<0.0001
Glucose (mmol/L) ^4^	5275	4.9 (2.1)	4.8 (1.0)	4.9 (1.2)	4.9 (1.5)	0.1
Energy (MJ/day)	16,814	7.63 (2.21)	7.87 (2.25)	7.97 (2.19)	8.26 (2.19)	<0.0001
Fibre (g/day)	16,814	20.1 (7.46)	21.3 (7.30)	21.6 (7.05)	21.9 (6.83)	<0.0001
Protein (g/day)	16,814	75.7 (22.7)	77.4 (21.7)	77.5 (20.6)	82.0 (21.9)	<0.0001
Total fat (g/day)	16,814	63.5 (23.9)	65.2 (24.1)	66.9 (23.1)	70.5 (23.6)	<0.0001
*n*-3 FA(g/day)	16,814	0.68 (0.72)	0.79 (0.64)	0.78 (0.62)	0.81 (0.69)	<0.0001
Saturated fat (g/day)	16,814	27.1 (11.1)	27.2 (10.7)	27.9 (10.3)	29.6 (10.3)	<0.0001
Alcohol (g/day) ^3^	16,814	4.2 (5.8)	3.5 (4.6)	3.1 (4.2)	2.8 (4.0)	<0.0001

^1^ *n* of total sample population (*n* = 23 907). Numbers of participants vary because of missing information variables; ^2^ *p*-value by analysis of variance (ANOVA) with fish consumption as categorical variable (never, <1, 1, 2–3 or more); ^3^ Alcohol intake (estimated from questionnaire) (g/day); ^4^ Blood samples are non-fasting. Abbreviations: BMI: Body Mass Index, WC: Waist circumference, HDL-C: high-density lipoprotein cholesterol, SBP: Systolic blood pressure, DBP: Diastolic blood pressure, FA: fatty acid. The Tromsø Study: Tromsø 4.

**Table 4:** Changes in metabolic score and components of metabolic syndrome from baseline to follow-up, mean (SD).

		Baseline	Follow-Up	Mean Difference	*p* ^1^
Metabolic score	Women	0.80 (0.01)	1.57 (0.02)	0.76 (0.02)	<0.0001
	Men	1.10 (0.01)	1.82 (0.02)	0.72 (0.02)	<0.0001
Waist circumference	Women	83.0 (0.3)	91.8 (0.2)	8.8 (0.2)	<0.0001
	Men	94.4 (0.2)	100.6 (0.2)	6.2 (0.2)	<0.0001
Triglycerides ^2^	Women	1.28 (0.01)	1.41 (0.02)	0.13 (0.01)	<0.0001
	Men	1.81 (0.02)	1.68 (0.02)	−0.13 (0.02)	<0.0001
HDL-C ^2^	Women	1.64 (0.01)	1.64 (0.01)	−0.01 (0.01)	0.30
	Men	1.33 (0.004)	1.35 (0.01)	0.02 (0.004)	<0.0001
Systolic blood pressure	Women	131.4 (0.2)	136.5 (0.4)	5.1 (0.3)	<0.0001
	Men	139.6 (0.2)	142.1 (0.3)	2.5 (0.3)	<0.0001
Diastolic blood pressure	Women	77.3 (0.2)	75.5 (0.2)	−1.8 (0.2)	<0.0001
	Men	82.0 (0.2)	81.7 (0.2)	−0.3 (0.2)	0.08
Glucose ^2^	Women	4.74 (0.03)	5.19 (0.02)	0.45 (0.03)	<0.0001
	Men	4.82 (0.03)	5.43 (0.03)	0.61 (0.03)	<0.0001

^1^ *p*-value derived from linear mixed model, adjusted for age with consumption of fatty fish, lean fish, processed fish, age and time in model. ^2^ Blood samples are non-fasting. HDL-C: HDL cholesterol. The Tromsø Study: Tromsø 4 and 6.

**Table 5:** Estimated change in metabolic score by consumption fish.

	Model	Women			Men		
		B	95% CI	*p*	B	95% CI	*p*
Fatty fish	1	0.03	−0.004 to 0.07	0.09	0.01	−0.03 to 0.05	0.7
	2	0.06	0.02 to 0.10	<0.01	0.02	−0.03 to 0.06	0.5
Lean fish	1	−0.05	−0.09 to −0.01	0.02	−0.10	−0.15 to −0.05	<0.0001
	2	−0.05	−0.09 to −0.00	0.05	−0.07	−0.13 to −0.02	<0.01
Processed fish	1	0.03	−0.002 to 0.07	0.06	0.01	−0.03 to 0.05	0.7
	2	0.03	−0.01 to 0.07	0.1	<−0.01	−0.04 to 0.04	1.0

Estimated change (regression coefficient B and 95% confidence interval) in metabolic score among those consuming fatty/lean/processed fish once a week or more, compared to those consuming fatty/lean/processed fish less than once a week. Metabolic score: A score from zero to five for each fulfilled feature of MetS (based on the JIS definition). Blood samples were non-fasting. Model 1: Age adjusted linear mixed model with consumption of fatty fish, lean fish, processed fish, age, and time covariates. Model 2: Multiple models with consumption of fatty fish, lean fish, processed fish, vitamin D use, cod liver oil/ fish oil capsules use, smoking, leisure physical activity, energy intake, alcohol intake, education, age and time in model. The Tromsø Study: Tromsø 4 and 6.

**Table 6:** Estimated change in waist circumference and blood pressure, by consumption of fish.

	Model	Women			Men		
		B	95% CI	*p*	B	95% CI	*p*
WC							
Fatty fish	1	0.97	0.29 to 1.65	<0.01	0.60	0.01 to 1.18	0.05
	2	1.60	0.80 to 2.40	<0.0001	0.99	0.32 to 1.65	<0.01
Lean fish	1	−0.22	−1.09 to 0.65	0.6	−1.15	−1.96 to −0.35	<0.01
	2	0.05	−0.93 to −1.03	0.9	−0.45	−1.34 to 0.44	0.3
Processed fish	1	0.004	−0.68 to 0.69	1.0	−0.09	−0.66 to 0.48	0.8
	2	0.06	−0.74 to 0.86	0.9	0.07	−0.57 to 0.71	0.8
SBP							
Fatty fish	1	0.32	−0.36 to 0.99	0.4	0.18	−0.49 to 0.85	0.6
	2	0.21	−0.55 to 0.96	0.6	0.04	−0.70 to 0.78	0.9
Lean fish	1	−0.44	−1.22 to 0.34	0.3	−0.86	−1.66 to −0.06	0.04
	2	−0.31	−1.15 to 0.53	0.5	−0.78	−1.64 to 0.08	0.08
Processed fish	1	0.62	−0.06 to 1.30	0.1	0.49	−0.15 to 1.13	0.1
	2	0.09	−0.66 to 0.83	0.8	0.19	−0.51 to 0.90	0.6
DBP							
Fatty fish	1	0.29	−0.17 to 0.75	0.2	0.10	−0.37 to 0.56	0.7
	2	0.37	−0.15 to 0.89	0.2	−0.10	−0.61 to 0.42	0.7
Lean fish	1	−0.23	−0.76 to 0.31	0.4	−0.63	−1.18 to −0.07	0.03
	2	−0.18	−0.76 to 0.40	0.5	−0.43	−1.04 to 0.17	0.2
Processed fish	1	0.20	−0.26 to 0.66	0.4	0.13	−0.31 to 0.57	0.6
	2	0.26	−0.25 to 0.78	0.3	0.09	−0.40 to 0.58	0.7

Estimated change (regression coefficient B and 95% confidence interval) in waist circumference and blood pressure among those consuming fatty/lean/processed fish once a week or more, compared to less than once a week. WC: Waist circumference, SBP: Systolic blood pressure, DBP: Diastolic blood pressure. Plasma glucose was non-fasting. Model 1: Age adjusted linear mixed model with consumption of fatty fish, lean fish, processed fish, age, and time covariates. Model 2: Multiple models with consumption of fatty fish, lean fish, processed fish, vitamin D use, cod liver oil/ fish oil capsules use, smoking, leisure time physical activity, energy intake, alcohol intake, education, age and time in model. The Tromsø Study: Tromsø 4 and 6.

**Table 7:** Estimated change in triglyceride, HDL-cholesterol and blood glucose, by consumption of fish.

	Model	Women			Men		
		B	95% CI	*p*	B	95% CI	*p*
Triglyceride							
Fatty fish	1	0.004	−0.03 to 0.04	0.8	−0.0003	−0.05 to 0.046	1.0
	2	0.001	−0.04 to 0.04	1.0	0.02	−0.03 to 0.07	0.5
Lean fish	1	−0.04	−0.08 to −0.00	0.04	−0.11	−0.17 to −0.06	<0.0001
	2	−0.03	−0.07 to 0.01	0.1	−0.11	−0.17 to −0.05	0.001
Processed fish	1	0.01	−0.021 to 0.05	0.5	0.01	−0.03 to 0.06	0.5
	2	0.02	−0.02 to 0.05	0.4	0.02	−0.03 to 0.07	0.4
HDL–C							
Fatty fish	1	0.01	−0.00 to 0.03	0.1	0.02	0.00 to 0.03	0.03
	2	0.001	−0.02 to 0.02	0.9	0.002	−0.01 to 0.02	0.8
Lean fish	1	0.03	0.01 to 0.05	<0.01	0.04	0.02 to 0.05	<0.0001
	2	0.02	0.00 to 0.04	0.05	0.03	0.01 to 0.05	0.005
Processed fish	1	−0.02	−0.04 to −0.01	0.01	−0.02	−0.04 to −0.01	0.001
	2	−0.02	−0.04 to −0.01	0.01	−0.02	−0.04 to −0.01	0.002
Glucose							
Fatty fish	1	0.04	−0.02 to 0.10	0.2	0.05	−0.03 to 0.12	0.2
	2	0.06	−0.00 to 0.12	0.05	0.04	−0.05 to 0.12	0.4
Lean fish	1	−0.04	−0.11 to 0.03	0.3	0.04	−0.07 to 0.14	0.5
	2	−0.03	−0.10 to 0.04	0.4	0.08	−0.03 to 0.20	0.2
Processed fish	1	0.01	−0.05 to 0.06	0.8	0.04	−0.00 to 0.15	0.05
	2	−0.01	−0.07 to 0.05	0.7	0.04	−0.05 to 0.12	0.4

Estimated change (regression coefficient B and 95% confidence interval) in waist circumference and blood pressure among those consuming fatty/lean/processed fish once a week or more, compared to consuming less than once a week. TG: Triglycerides, HDL-C: HDL cholesterol. Plasma glucose was non-fasting. Model 1: Age adjusted linear mixed model with consumption of fatty fish, lean fish, processed fish, age, and time covariates. Model 2: Multiple models with consumption of fatty fish, lean fish, processed fish, vitamin D use, cod liver oil/ fish oil capsules use, smoking, leisure time physical activity, energy intake, alcohol intake, education, age and time in model. The Tromsø Study: Tromsø 4 and 6.
